# A mandatory role of nuclear PAK4-LIFR axis in breast-to-bone metastasis of ERα-positive breast cancer cells

**DOI:** 10.1038/s41388-018-0456-0

**Published:** 2018-09-03

**Authors:** Yanshu Li, Hongyan Zhang, Yue Zhao, Chunyu Wang, Zhenguo Cheng, Lina Tang, Yunling Gao, Furong Liu, Jiabin Li, Yan Li, Yang Li, Nanxi Geng, Xue Rui, Yuee Teng, Yunpeng Liu, Liu Cao, Rakesh Kumar, Feng Jin, Feng Li

**Affiliations:** 1 Department of Cell Biology, Key Laboratory of Cell Biology of Ministry of Public Health, and Key Laboratory of Medical Cell Biology of Ministry of Education, China Medical University, No. 77, Puhe Road, Shenyang North New Area, 110122 Shenyang, Liaoning China; 2 Department of Surgical Oncology, The First Hospital of China Medical University, No. 155, North Nanjing Street, Heping District, 110001 Shenyang, Liaoning, China; 3 Department of Medical Oncology, The First Hospital of China Medical University, No. 155, North Nanjing Street, Heping District, 110001 Shenyang, Liaoning, China; 4 Cancer Research Program, Rajiv Gandhi Centre for Biotechnology, Thiruvananthapuram, 695014 Kerala India

**Keywords:** Breast cancer, Cell migration, Hormone receptors

## Abstract

The mechanism of estrogen receptor alpha (ERα)-positive breast cancer-associated bone metastasis is poorly understood. In this article, we report that nuclear p21-activated kinase 4 (nPAK4) is a novel repressor of ERα-mediated transactivation in a 17β-estradiol (E2)-dependent manner and promotes PAK4–ERα axis-mediated bone metastasis by targeting leukemia inhibitory factor receptor (LIFR) in ERα-positive breast cancer. An evaluation of clinical breast cancer samples revealed that nPAK4 is linked to ERα expression and appears to be associated with a poor prognosis in bone metastatic breast cancer. PAK4 bound and co-translocated with ERα from the cytoplasm to the nucleus upon stimulation with E2. nPAK4 enhanced the invasive potential of ERα-positive breast cancer cells in vitro and promoted breast cancer metastasis in vivo. Mechanistically, nPAK4 promoted the metastasis of ERα-positive breast cancer cells by targeting LIFR, a bone metastasis suppressor. Strikingly, the nuclear accumulation of PAK4 might promote aggressive phenotypes, highlighting nPAK4 as a novel predictive biomarker for ERα-positive breast cancer bone metastasis.

## Introduction

Breast cancer is the second leading cause of cancer death among females worldwide [1], and there is an increasing trend of breast cancer affecting women younger than 45 years of age [2]. About 75% of breast cancers are estrogen receptor-alpha positive (ERα+) [3] and associated bone metastasis causes a significant morbidity and mortality in the late-stage breast cancer patients [4]. Currently, our understanding of dysregulated pathways with role in both transformation and directional motility, as essential component of the productive metastasis, remains poorly understood, and there is currently no effective therapy to extend the survival of patients with bone metastasis [5].

The p21-activated kinase 4 (PAK4) oncogene is amplified and/or overexpressed in a large variety of human cancers [6–10], including, breast cancer [11–14]. In addition, PAK4 status is a strong prognostic factor for relapse and poor overall survival [15] in breast cancer patients [13, 16]. At the cellular level, PAK4 signaling regulates a number of cellular pathways with roles in transformation, cytoskeletal organization, cell motility, and cell cycle regulation [17]. However, the role of the nuclear PAK4 (nPAK4) signaling in breast cancer metastasis is largely unknown. Here, we provide evidence that nPAK4 is an effective repressor of ligand-induced estrogen receptor alpha (ERα) transcriptional activity. In addition, we found that nPAK4-ERα axis contributes to breast-to-bone metastasis in ERα+ breast cancer via antagonizing the activity of a breast cancer bone metastasis suppressor, leukemia inhibitory factor receptor (LIFR) [18, 19]. And physiological significance of these mechanistic observations is supported by the finding that nPAK4 status in ERα + human breast cancer is closely associated with bone metastasis and a poor prognosis of a subset of breast cancer patients.

## Results

### Elevated nuclear PAK4 expression associates with bone metastasis and poor clinical outcomes of ERα+ breast cancer

**Figure 1:**
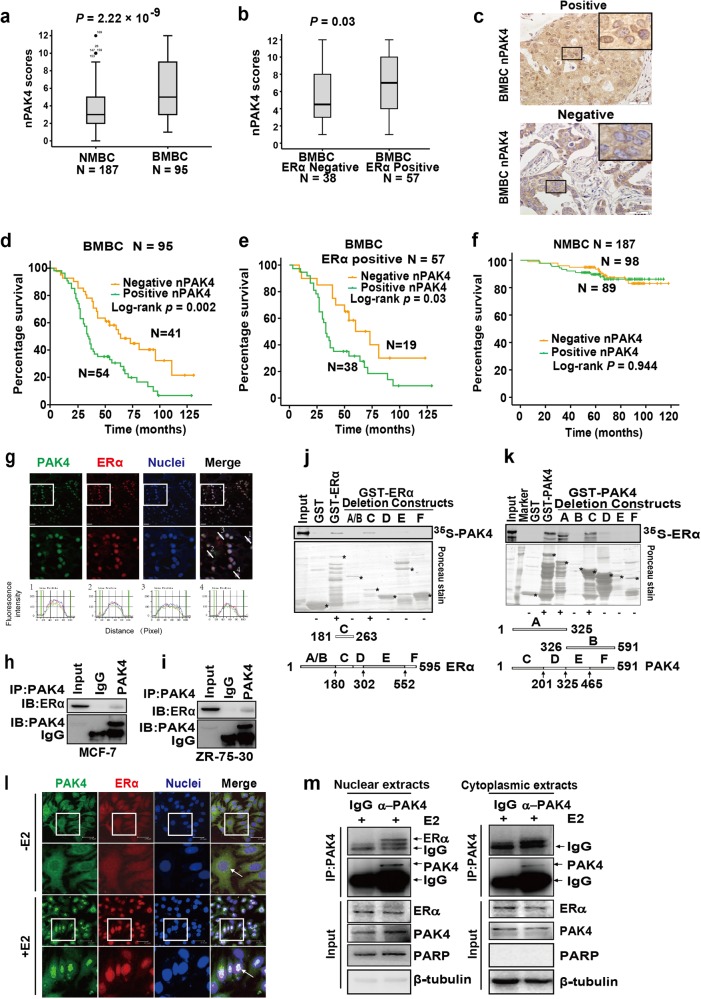
Nucleus PAK4 is a malignant effector in ERα+ breast cancer with bone metastasis. **a** Box plot of nPAK4 in the NMBC or BMBC patients. The subjects with breast cancer were divided into two groups based on bone metastasis. **b** Box plot of nPAK4 in BMBC samples from 95 subjects. The subjects were divided into two groups based on ERα expression scores in the tumors, representing negative and positive for ERα expression. The data were analyzed using the Mann–Whitney *U* test. The horizontal lines represent the median; the bottom and top of the boxes represent the 25th and 75th percentiles, respectively, and the vertical bars represent the range of the data. **c** Two representative images showing positive (upper picture) or negative (lower picture) nPAK4 localization in the BMBC samples. Scale bars, 50 µm. **d**, **e** Ninety-five cases of BMBC and 57 cases of ERα + BMBC were divided into two groups using the nPAK4 localization signal. The relationship between nPAK4 protein expression and bone metastasis-free survival (BMFS) was analyzed according to the Kaplan–Meier method. *P* values were obtained using the log-rank test. **f** PAK4 expression in the nucleus of breast cancer cells was not significantly associated with non-bone relapses (brain, liver, or lung). Kaplan–Meier survival analysis of 187 patients with breast cancer separated into two groups based on the median value of the nPAK4 localization signal. The positive group is shown in green (*n* = 89), and the negative group is shown in orange (*n* = 98). *P* values were calculated using the log-rank test. **g** Representative images of ERα+ breast cancer tissue (green, PAK4; red, ERα; and blue, nuclei). Scale bar, 20 μm. The 2nd lines are the 2.5-folds enlarged pictures of the 1st lines, respectively. The image-pro plus 6.0 software convert immunofluorescence staining into peaks/curves at a 3rd line across the image. MCF-7 **h** and ZR-75-30 **i** cell lysates were immunoprecipitated with PAK4 antibodies or IgG. Then, endogenous ERα and PAK4 were detected using immunoblot assays. **j**, **k** For the GST pull-down assay, GST, GST-ERα, GST-PAK4 plus GST-ERα deletions or GST-PAK4 deletions were incubated with the indicated proteins, transcripted, and then translated in vitro. Bound proteins were detected with auto-radiography. A schematic representation of the ERα and PAK4 deletion constructs is shown. **l** Representative PAK4 and ERα immunostaining in MCF-7 cells treated with or without E2 (10^−9^ M). PAK4 (green); ERα (red); and nuclei were stained with DAPI (blue). Merged images are shown as indicated. Original magnification: × 40. Scale bar: 37.5 μm. **m** Co-IP of PAK4 and ERα from the nuclear and cytoplasmic fractions obtained from human MCF-7 cells treated with E2 (10^−9^ M) for 45 min. β-tubulin and PARP were used as controls for the cytoplasmic and nuclear compartments, respectively

To investigate the significance of PAK4 in the pathobiology of breast cancer, we evaluated the status and subcellular localization of PAK4 using immunohistochemical staining in 187 cases of non-bone metastatic breast cancer (NMBC) and 95 cases of bone metastatic breast cancer (BMBC) specimens with a long-term clinical follow-up. We found that the status of the nuclear PAK4 (nPAK4) scores were significantly higher in the BMBC group than in the NMBC group (*P* = 2.22 × 10^−9^) (Fig. 1a). Moreover, we found that nPAK4 expression in the BMBC group exhibited a significant positive correlation with ERα but no significant correlation with the status of the progesterone receptor (PR), Her2 or Ki-67 (Supplementary Table 1). To better understand the correlation of nPAK4 with ERα in the BMBC group, we divided the BMBC samples into two groups based on the amount of ERα as defined by expression scores. In tumors with nPAK4, positivity for ERα expression was greater than in those that were negative for ERα expression (Fig. 1b; *P* = 0.03). In contrast, high nPAK4 but low ERα expression levels were detected in the NMBC tissues with an inverse correlation between these two groups (Supplementary Table 2; *P* = 0.0032). These findings suggest that nPAK4 may function as a bone metastasis-associated protein in ERα+ breast cancer.

PAK4 is overexpressed in primary human breast cancer and breast cancer cell lines, and the upregulation of PAK4 may be an important event in tumorigenesis that contributes to progression and metastasis. Representative images from BMBC specimens that were nPAK4-positive and nPAK4-negative are shown in Fig. 1c. We next performed Kaplan–Meier analyses to determine whether nPAK4 is a prognostic marker for clinical outcome. Ninety-five BMBC patients were analyzable for bone metastasis-free survival (BMFS), and 54 cases (56.8%) are positive nPAK4 expression, whereas 41cases (43.2%) are negative nPAK4 expression. The patients with nPAK4-positive tumors had shorter BMFS times (47.5 ± 4.7 months, mean ± s.e.m.) than those who had tumors negative for nPAK4 expression (73.5 ± 7.1 months; *P* = 0.002; Fig. 1d). Among the 95 BMBC patients, 57 BMBC patients were ERα-positive expression (Supplementary Table 1). Then, we divided 57 BMBC patients into two groups according to the nPAK4-positive or -negative expression. The patients with nPAK4-positive tumors (*n* = 38, 48.6 ± 6.0 months, mean ± s.e.m.) had shorter BMFS times than those with nPAK4-negative tumors (*n* = 19, 71.7 ± 9.7 months; *P* = 0.03; Fig. 1e). Notably, the impact of nPAK4 expression on prognosis was not observed in the NMBC group (*P* = 0.944; Fig. 1f). To evaluate the impact of nPAK4 and pathological factors on the prognosis of the BMBC patients, we performed univariate and multivariate analyses using the Cox proportional hazards model. The univariate analysis of the BMFS rate revealed statistically significant variable: positivity for the nuclear expression of PAK4 (*P* = 0.002). In the multivariate analyses, positivity for the nuclear expression of PAK4 (*P* = 0.003) was associated with a poor BMFS rate (Supplementary Table 3). Collectively, these data suggest the potential use of nPAK4 in the prognostic stratification of patients with ERα + BMBC.

### PAK4 associates with ERα

To investigate the relationship between PAK4 and ERα, ERα+ breast cancer tissues were analyzed for the status of PAK4 via immunofluorescence staining. We noticed a remarkable co-localization of PAK4 and ERα was observed in the nucleus in the ERα+ breast cancer tissue and the white lines in Fig. 1g were converted immunofluorescence staining into peaks/curves using the Line Profile Tool of Image-Pro Plus 6.0. (Fig. 1g). To investigate the significance of these physiologically relevant setting, we used ERα+ MCF-7 and ZR-75-30 cells as a model system in the subsequent studies. Results from co-immunoprecipitation (co-IP) studies demonstrated that the endogenous PAK4 associates with endogenous ERα in both MCF-7 and ZR-75-30 cells (Fig. 1h, i, respectively).

We next defined the regions of PAK4 and ERα that are required for the noted interaction between these proteins. The glutathione S-transferase (GST) pull-down assay showed that the N terminus (1–201 aa) of PAK4 and the DNA-binding domain (181–263 aa) of ERα are indispensable for the observed PAK4–ERα interaction (Fig. 1j, k). In general, PAKs have been recognized as a downstream regulator of the Rho family GTPases [20] where PAK’s kinase activity plays a central role in the cellular outcome. However, we found that PAK4-mediated ERE transcriptional activation did not require kinase activity (Supplementary Figure S1). These results demonstrated that PAK4 associated with ERα, and the immunofluorescence studies indicated that PAK4 co-translocated with ERα from the cytoplasm to the nucleus upon 17β-estradiol (E2) treatment of MCF-7 cells (Fig. 1l). Furthermore, results from cell fractionation studies indicated the existence of PAK4/ ERα complex in the nuclear but not cytoplasmic compartment on MCF-7 cells stimulated with E2 (Fig. 1m), whereas, no physical interactions were detected between PAK4 and ERα in the MCF-7 without E2 stimulation (Supplementary Figure S2). We consider that ERα undergoes major conformational changes, resulting in receptor dimerization upon E2 binding, PAK4/ERα/E2 complex will instantly enter the nucleus and that is why we did not detect the binding between PAK4 and ERα with E2 stimulation in MCF-7 cytoplasmic extracts by co-IP assay. (Fig. 1m, Cytoplasmic extracts). These results suggest that ERα directly interacts with PAK4, PAK4 and ERα co-translocate into the nucleus in the presence of E2, and PAK4 may play a crucial role in steroid-hormone-mediated signal transduction.

### PAK4 is a novel corepressor of ligand-dependent ERα transactivation

**Figure 2:**
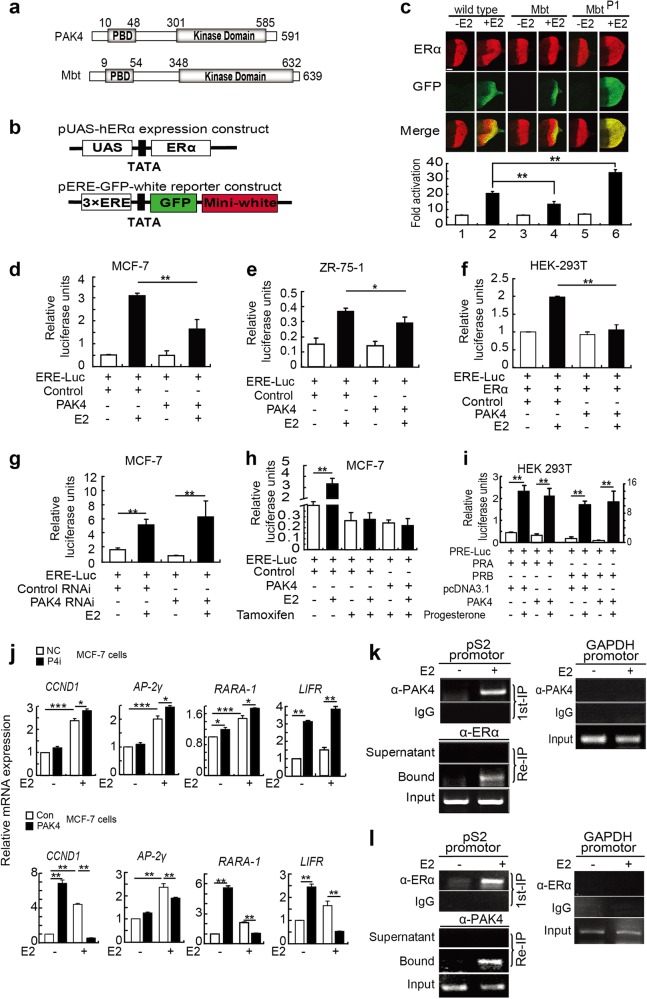
PAK4 represses ERα-mediated transactivation in an E2-dependent manner. **a** Schematic representation of *Drosophila* Mbt and its ortholog in human, PAK4. The PBD and Kinase domain are positioned in the Mbt and PAK4 proteins. Numbers indicate amino-acid position. **b** Schematic representation of the expression and reporter constructs. The expression constructs include human ERα driven by a *UAS* promoter. The reporter construct harbors the *GFP* reporter gene controlled by the three copies of the *EREs* and the *white* reporter gene driven by the corresponding endogenous promoter. **c** Mbt represses ERα-induced transactivation in *Drosophila*. Fly lines carrying a gain-of-function mutation of Mbt (*GMR-G4*/ + ; *UAS-Mbt*/ + ) or a loss-of-function mutation of Mbt (*GMR-G4*/ + ; *Mbt*^*P1*^) were crossed to two fly models expressing ERα proteins and harboring an *ERE-GFP-white* reporter gene in the pericentric region. The expression of ERα controlled by a *GMR-GAL4* driver in the *UAS-GAL4* system in eye imaginal discs of the third instar larvae was assessed by immunostaining using an anti-ERα antibody (upper panels). The effects of the Mbt and Mbt^P1^ mutations on ERα-mediated transactivation were assessed by examining of GFP expression (middle panels). Merged images are shown in the lower panels. The quantification of GFP expression as revealed by color intensity gradations with Adobe Photoshop (histogram) is shown at the bottom. **P < 0.01. Scale bars, 50 μm. **d**, **e** The transcriptional activity of ERα was decreased by overexpression of PAK4 both in MCF-7 and ZR-75-1 cells. Cells were transiently transfected with pGL3-ERE-Luc (ERE-Luc), control and PAK4 expression plasmids as indicated in the absence or presence of ligand (E2). **f** The ERE luciferase reporter assay in HEK 293T cells were transfected with the expression vector encoding ERα and the indicated constructs. **g** Control RNAi and PAK4 RNAi were transfected into MCF-7 cells, and 24 h later, the cells were transiently transfected with pGL3-ERE-Luc. All the transfected cells were treated with or without E2 (10^−9^ M) for 24 h. **h** ERE luciferase activity in MCF-7 cells transfected with ERE-Luc and the PAK4 and control expression vectors. Cells were treated with or without 10^−9^ M E2 and treated with 10^−6^ M tamoxifen for 24 h before harvesting. **i** PAK4 does not affect progesterone-induced PR transcriptional activation. HEK 293T cells were co-transfected with a PRE-Luc reporter construct and an expression vector encoding PRA or PRB and PAK4. Cells were treated with or without 10^−8^ M progesterone for 24 h before harvesting. All the data are represented as the means ± s.e.m. **P *<* 0.01; *P *<* 0.05. **j** Real-time quantitative PCR (RT-qPCR) analysis showing the effect of PAK4 on activation of four ERα target genes. MCF-7 cells stably overexpressing or knocking down PAK4 were harvested after treatment with or without E2 (10^−9^ M) for 24 h. Total RNA was analyzed by RT-qPCR. Levels of all mRNAs were normalized to that of β-actin mRNA. Statistical significance of differences between experimental groups was assessed one-way ANOVA. Error bars represent mean ± S.E.M. *P < 0.05, **P < 0.01, ***P < 0.001. **k**, **l** PAK4 and ERα are predominantly recruited to the ERE at heterochromatic loci in the presence of E2. MCF-7 cells were grown in medium with or without E2 (10^−9^ M) for 45 min. ChIP assays and ChIP/Re-IP experiments were performed using specific antibodies against PAK4 and ERα, as indicated

To delineate the potential effect of PAK4 in ERα transactivation function, we followed a genetic approach in *Drosophila* [21], which involves the use of *Mbt* and the corresponding loss-of-function of mutation *Mbt*^*P1*^ (a gift of Dr. Raabe T.) [22] as *Drosophila mushroom bodies tiny* (*Mbt*) shares considerable sequence homology with the p21-binding domain (PBD) and kinase domain of mammalian PAK4 (Fig. 2a). The transactivation function of ligand-activated nuclear receptors is mediated by the interplay of coregulators and coregulatory complexes [23, 24]. We determined that upon E2 stimulation, PAK4 and ERα co-translocate to the nucleus. Next, we hypothesized that PAK4 may be a new coregulator of ERα-induced transactivation. We analyzed the effects of *Mbt* on ERα-dependent gene activation in vivo by testing ERα-mediated transactivation using the green fluorescent protein (GFP) reporter gene expression system. In this system, ERα was expressed in the *Drosophila* eye using the *glass multimer reporter* (*GMR*) gene promoter. A reporter construct containing the white gene and a gene encoding the GFP controlled by three estrogen response elements (3 × *ERE*) was inserted into a heterochromatic region leading to a mosaic red eye phenotype (Fig. 2b). Ectopic expression of the wild-type, *Mbt* or mutant *Mbt*^*P1*^, together with GFP reporter gene, allowed us to demonstrate that ERα expressed in the fly tissues was transcriptionally functional. We found that indeed, *Mbt* significantly reduced the ligand-induced ERα transactivation function (*Mbt* drives expression of destabilized GFP fluorescence), whereas *Mbt*^*P1*^ dominantly increased GFP transactivation by ERα (*Mbt*^*P1*^ drives expression of enhanced GFP fluorescence) (Fig. 2c). In brief, *Mbt* inhibits ERα transactivation in an E2-dependent manner in the *Drosophila* eye model.

To investigate whether PAK4 could also inhibit ERα-induced transactivation, a series of promoter-luciferase assays were performed in human cell lines. We found that PAK4 inhibits E2-stimulated, ERα-mediated transactivation activity in a substantial manner (Fig. 2d−f). Consistent with this observation, the silencing of PAK4 expression in MCF-7 cells also resulted in an enhancement of *ERE*-driven transactivation function of ERα, suggesting that PAK4 represses ERα transactivation (Fig. 2g). To confirm PAK4 as a corepressor by recruiting histone deacetylase (HDAC), the co-immunoprecipitation for exogenous and endogenous interaction were detected in HEK 293T and MCF-7 cell lines, respectively. The results indicate that PAK4 interacts with HDAC1 (Supplementary Figure 3a, b). Interestingly, Mbt overexpression promotes HDAC1 recruitment to its target chromatin (Supplementary Figure 3c, d). PAK4 may repress the ERα transactivation function as an integral component of HDAC-containing corepressor complexes. Furthermore, noted PAK4 induced the repression of ERα transcription activation could be effectively blocked by tamoxifen, an estrogen receptor antagonist (Fig. 2h). Notable, PAK4 did not influence the transactivation of progesterone receptor A/B (PR A/B) (Fig. 2i). These observations suggest that PAK4 represses ERα-mediated transactivation in an E2-dependent manner.

Having established that PAK4 downregulates ligand-induced *ERE*-driven transcriptional activity, we next examined whether PAK4 is required for regulating the endogenous ERα target genes. Using real-time quantitative polymerase chain reaction, we assessed the role of PAK4 in the endogenous ERα-responsive genes. Similar to what we observed in luciferase assay, PAK4 resulted in a significant decrease in the E2-mediated expression of four ERα target genes, including CCND1, AP-2γ, RARA-1, and LIFR in MCF-7 cells (Fig. 2j). we next investigated whether PAK4/ERα complex is recruited on ERα target gene, the *pS2* chromatin. ChIP assays were first performed with antibodies against PAK4 or ERα using the soluble chromatin derived from MCF-7 cells in the presence or absence of E2 (Fig. 2k−l, upper panel). We found that indeed, PAK4 and ERα were co-recruited onto the *pS2* gene chromatin (at position −3181 to −3062). Collectively, these findings suggested that PAK4 may be a negative regulator of ERα transactivation in ERα+ breast cancer cells, which may contribute to the progression of breast cancer by allowing the development of ERα-negative phenotypes, leading to increased aggressiveness in ERα+ breast cancer cells.

### Nuclear PAK4 reverses E2-mediated gene expression in mammalian cells

**Figure 3:**
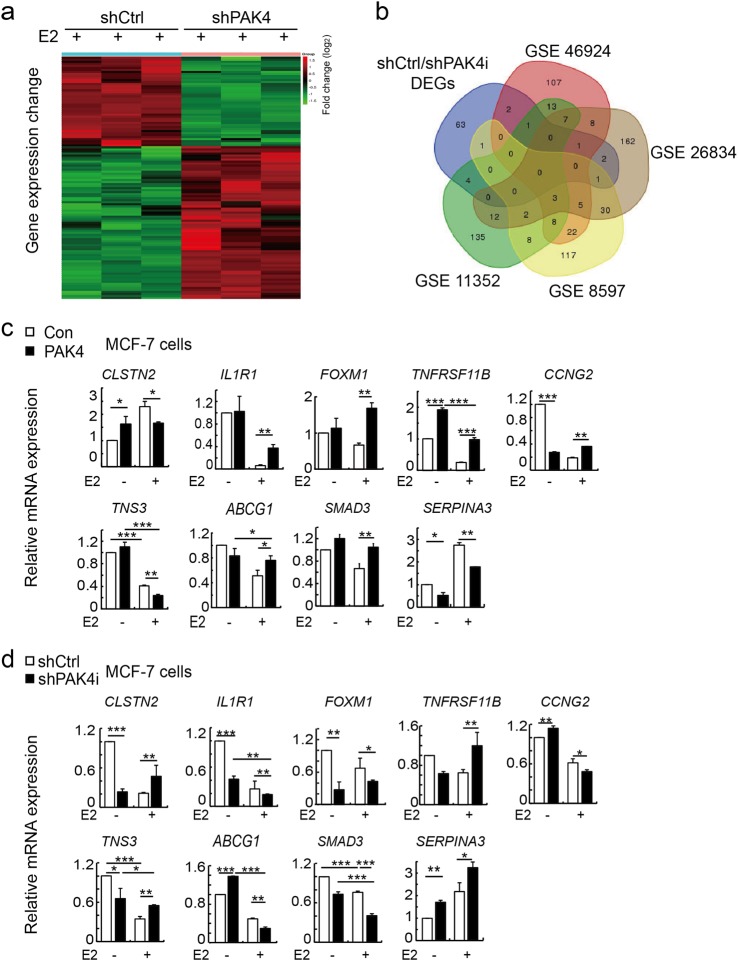
PAK4 reverses ERα-mediated gene expression. **a** RNA-seq analysis profile of shPAK4 or shCtrl after treatment with E2 in MCF-7 cell. Heat map displays the altered expression of E2-induced genes upon PAK4 knockdown. Fold change is indicated at right. **b** Venn diagram showing four sets of microarray data from GEO databases (GSE46924, GSE26834, GSE8597, and GSE11352) and shCtrl/shPAK4 differentially expressed genes. **c**, **d** Real-time PCR (RT-PCR) analysis showing overexpression or knockdown of PAK4 reverses nine of ERα-mediated gene expression. MCF-7 cells stably overexpressing PAK4 (**c**) or stably knocking down PAK4 (**d**) were harvested after treatment with or without E2 (10^−9^ M) for 24 h. Total RNA was analyzed by Real-time quantitative PCR (RT-qPCR). Error bars represent mean ± s.d. *P < 0.05; **P < 0.01; ***P < 0.001

Our results demonstrated that nPAK4 significantly repressed ERα-mediated transactivation in the presence of E2. Then, we continued to explore whether PAK4 was able to regulate E2-mediated target genes in mammalian cells, RNA sequencing (RNA-seq) analysis was used to detect differentially expressed genes (DEGs). From the gene expression data, we identified 75 DEGs in knockdown of PAK4 *vs* control cells after treatment with E2 in MCF-7 cells (Fig. 3a). Subsequently, four sets of previously published microarray data (GSE46924, GSE26834, GSE8597, and GSE11352) were obtained from the GEO databases. We used top 250 DEGs of the four GEO databases, which were regulated by E2. To further screening the nPAK4 and E2-mediated target genes, the overlapping target genes were identified using Venn diagram (Fig. 3b and Supplementary Table 4). Using quantitative PCR (qPCR), we assessed the role of PAK4 in E2- ERα signaling. Overexpression or knockout of PAK4 in MCF-7 cells resulted in a significant reverse in the E2-mediated expression of nine target genes, including *CLSTN2*, *IL1R1*, *FOXM1*, *TNFRSF11B*, *CCNG2*, *TNS3*, *ABCG1*, *SMAD3*, and *SERPINA3* (Fig. 3c, d). We also got a significant increase in the E2-mediated expression of five target genes, including *CDKN3*, *ASCL1*, *SDK2*, *ATF3*, and *CREBBP* in MCF-7 cell lines and the molecular mechanism will be verified in PAK4/mammary gland mouse model in future (Supplementary Figure S4).

### Nuclear PAK4 promotes epithelial–mesenchymal transition (EMT) of ERα+ breast cancer cells

**Figure 4:**
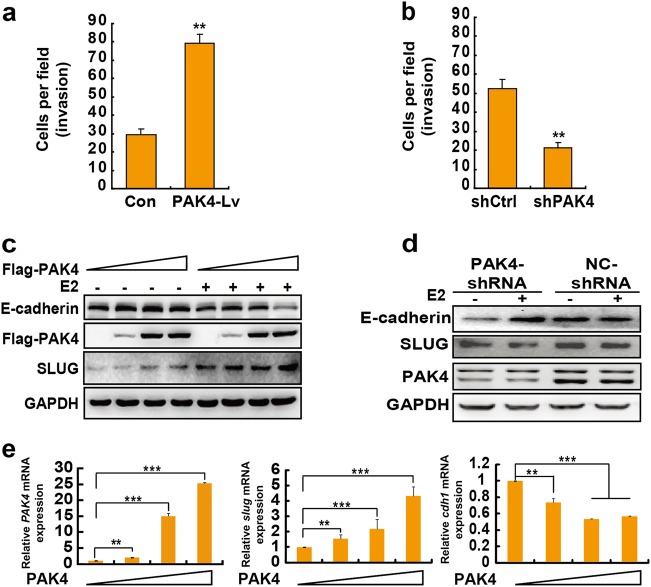
Nuclear PAK4 promotes breast cancer cell invasion via downregulation of E-cadherin. **a** PAK4 promotes the invasion of breast cancer cells with E2 stimulation. Transwell invasion assays of MCF-7 cells that were infected with lentivirus Con or PAK4. The number of cells was counted in 16 independent symmetrical microscopic visual fields (× 400 original magnification). The bar chart shows values of the means ± s.d. from three independent experiments. **P < 0.01. **b** Knockdown of PAK4 suppresses the invasion of human breast cancer cells with E2 stimulation. Transwell invasion assay of shCtrl or shPAK4 MCF-7 cells. The data are shown as the means ± s.d. from triplicate experiments. **P < 0.01 according to Student’s *t*-test. **c** PAK4 regulates E-cadherin and Slug protein expression in breast cancer cells. MCF-7 cells were transiently transfected with control or increasing amounts of Flag-PAK4 expression plasmid with or without E2 (10^-9^ M) for 48 h. E-cadherin, Slug and PAK4 were detected by western blotting. GAPDH was used as a loading control. **d** MCF-7 cells infected with shPAK4 lentivirus or control shRNA (shCtrl) for 3 d and were either untreated or treated with E2 for 24 h. Western blot analysis revealed PAK4, E-cadherin and Slug protein levels. **e** PAK4 overexpression resulted in the suppression of *cdh1* and promotion of *slug* gene expression. MCF-7 cells were transiently transfected with control or increasing amounts of Flag-PAK4 expression plasmids and treated with E2 (10^−9^ M) for 24 h. The cells were then harvested and analyzed for *PAK4*, *slug*, and *cdh1* mRNA using quantitative real-time PCR assays. Real-time PCR values were normalized to the housekeeping gene β-actin. Experiments were performed three times, each with technical duplicates in the quantitative RT-PCR assays, and the data are presented as the means ± s.d. **P < 0.01; ***P < 0.001 according to Student’s *t*-test

We next analyzed the role of nPAK4 in conferring invasive phenotypes to breast cancer cells using approaches involving the loss or gain of PAK4 functions by using knockdown (shPAK4) and overexpression (PAK4-Lv) approaches. The ability of non-invasive MCF-7 cells to invade through the Matrigel was markedly enhanced by PAK4 overexpression (Fig. 4a). Accordingly, we found that breast cancer cells with a depleted PAK4 were less efficient in migrating across the membrane as compared with the control cells with functional PAK4 (Fig. 4b). The overexpression of PAK4 led in a dose-dependent manner to a reduction of epithelial markers E-cadherin and the induction of the mesenchymal marker Slug in MCF-7 cells upon E2 stimulation (Fig. 4c, d). In contrast, as shown in Fig. 4e, the depletion of PAK4 in ERα+ ZR-75-1 cells led to a significant reduction in Slug and an increase in E-cadherin. These results suggest that PAK4 promotes a cellular EMT in an E2-dependent manner in the non-metastatic ERα+ breast cancer cells.

### Nuclear PAK4 targets LIFR to breast-to-bone metastasis of ERα+ breast cancer cells

**Figure 5:**
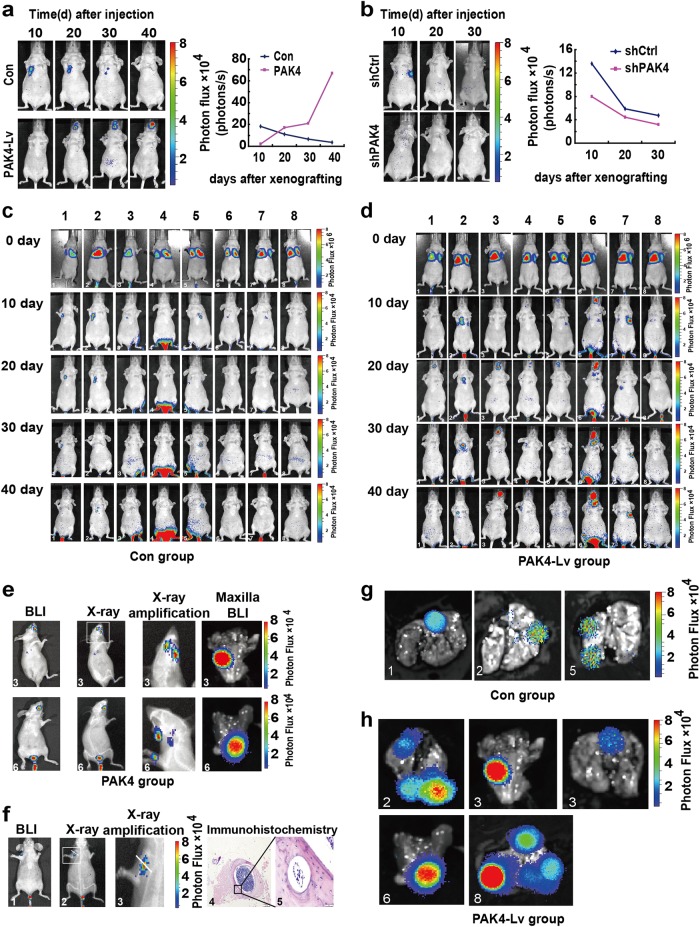
Nuclear PAK4 induces ERα+ breast cancer metastasis to the bone. MCF-7-Luc-F5 cells were infected with lentiviruses carrying either a control vector (Con), PAK4 expression construct (PAK4-Lv), control shRNA (shCtrl), or PAK4 shRNA (shPAK4), and were injected into tail vein of 6-week-old female SCID mice. **a**, **b** Representative in vivo BLI images of animals at the indicated time points are shown. Normalized BLI signals of metastases of mice injected intravenously with the indicated transfected cells. **c**, **d** Representative BLI images of eight animals after tail vein injection of Con and PAK4-overexpressing MCF-7-Luc-F5 cells at the indicated time points. **e**, **f** The effect of PAK4 on seeding bone metastases in mice intravenously injected with breast cancer cells. BLI and X-ray images and BLI of bone metastatic tissues from mice injected with MCF-7-Luc-F5 breast cancer cells transfected with a PAK4 overexpression vector. Bone sections from PAK4-overexpressing mice were stained with H&E. **g**, **h** Representative BLI images showed tumor growth primarily within the lung and bone metastases from Con group and PAK4-Lv group

To gain an insight of the effect of nPAK4 on tumor progression, a BALB/c nude mice model derived from MCF-7-luc-F5 cells was employed. MCF-7-luc-F5 cells were infected with a lentivirus harboring PAK4 (PAK4-Lv) or a control (Con), and estradiol valerate (E2V) was daily administered in the animals by oral gavage. As depicted in Fig. 5a, the group of mice injected with PAK4-Lv, but not control group, exhibited metastasis of cancer cells to the bones as measured by bioluminescence imaging (BLI) to quantify the photon flux. Following the injection of tumor cells into the lateral tail vein, BLI revealed that MCF-7-luc-F5 cells formed metastatic foci in the maxilla. In contrast, the knockdown of PAK4 did not affect the bone metastasis of cells in vivo (Fig. 5b).

In these experiments, cells were injected into the lateral tail vein (*n* = 8) of 6-week-old female BALB/c nude mice (~ 20 g). The dissemination of tumors was monitored every 10 days by BLI with an IVIS 200 imaging system. BLI confirmed that PAK4 overexpression resulted in markedly greater metastatic spread to the bone in these mice compared with that observed in the control group. Representative IVIS images of the PAK4-Lv and control groups on day 0 to day 40 are depicted in Fig. 5c, d, respectively. Six of the eight mice in the PAK4-Lv group exhibited bone tumorigenicity, whereas in the control group, there was no metastatic affinity for the bone. The metastasis of breast cancer to the maxilla and humerus was verified by BLI and X-ray examination (Fig. 5e−h), and metastasis to the bone was verified by hematoxylin and eosin staining (H&E stain) (Fig. 5f, picture 4 and 5). Taken together, the data suggest that PAK4 overexpression in a low metastatic potential ER+ breast cancer cells induced metastatic bone colonization in vivo.

**Figure 6:**
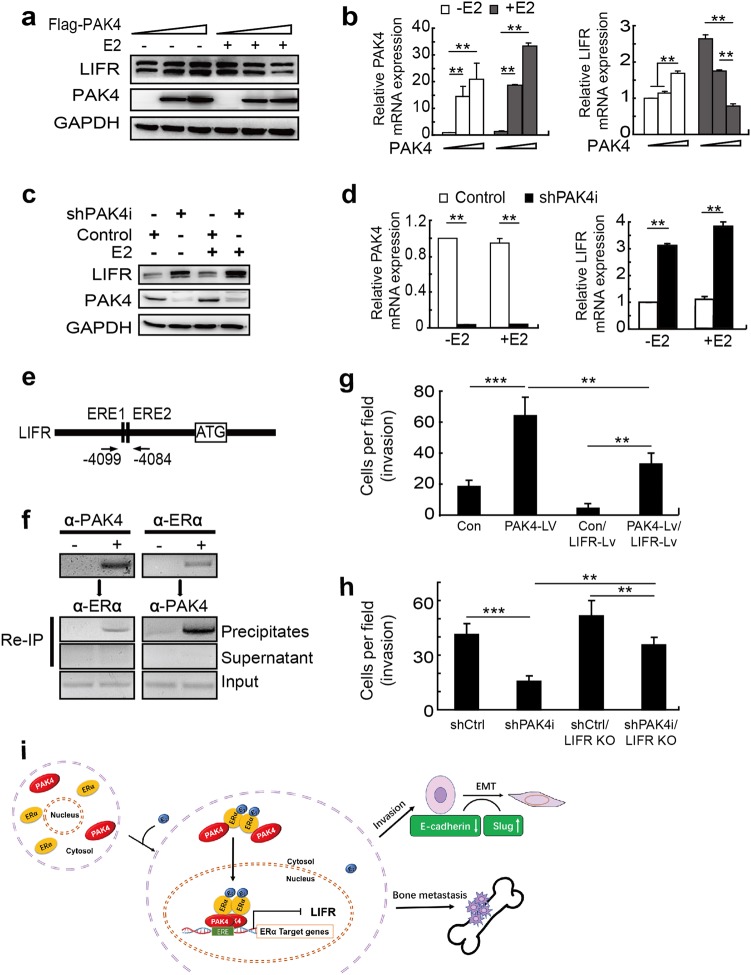
Nuclear PAK4-LIFR Axis involved in breast-to-bone metastasis of ERα-positive breast cancer cells. **a** PAK4 regulates LIFR protein in breast cancer cells. ZR-75-30 cells were transiently transfected with control or increasing amounts of Flag-PAK4 expression plasmids with or without E2 (10^-9^ M) for 48 h. LIFR and PAK4 were detected by western blot analysis. GAPDH was used as a loading control. **b** MCF-7 cells infected with shPAK4 lentivirus or control shRNA (shCtrl) for 3 d and were either untreated or treated with E2 for 24 h. A western blot revealed PAK4 and LIFR protein levels. **c** PAK4 resulted in the suppression of *LIFR* gene expression. MCF-7 cells were transiently transfected with control or increasing amounts of Flag-PAK4 expression plasmid and treated with E2 (10^−9^ M) for 24 h. The cells were then harvested and analyzed for *PAK4* and *LIFR* mRNA using quantitative real-time PCR assay. **d** MCF-7 cells infected with shPAK4 lentivirus or control shRNA (shCtrl) for 3 d and were either untreated or treated with E2 for 24 h. Real-time PCR assays revealed the *PAK4* and *LIFR* mRNA levels. The real-time PCR values were normalized to the housekeeping gene *β-actin*. Experiments were performed three times, each with technical duplicates in the quantitative RT-PCR assay, and the data are presented as the means ± s.d. *P* < 0.05 according to Student’s *t* test. **e**, **f** PAK4 and ERα are predominantly recruited to the *ERE* of *LIFR* in the presence of E2 in MCF-7 cells. ChIP/re-ChIP experiments were performed using specific antibodies against PAK4 and ERα as indicated. **g**, **h** Number of invading cells (mean ± s.d.) from three indicated experiments in the presence of E2. **P < 0.01, ***P < 0.001 according to one-way ANOVA. **i** Schematic representation of PAK4 corepressor functions in ERα-induced transactivation

Recently, one of the ERα downstream target gene, LIFR has been shown to suppress breast cancer bone metastasis [19]. However, the role of LIFR in the noted PAK4 regulation of breast-to-bone metastases remained unknown. In this context, we discovered that PAK4 overexpression remarkably inhibited the expression of the LIFR protein and mRNA following the E2 treatment of ZR-75-30 cells compared with the control cells (Fig. 6a, b). However, a PAK4 knockdown led to the upregulation of the LIFR protein and mRNA in MCF-7 cells with or without E2 treatment (Fig. 6c, d). Furthermore, we found that a PAK4–ERα complex is recruited onto ERα target gene (LIFR) chromatin. As expected, the Re-Chip assays indicated that PAK4 and ERα were recruited to two ERE half sites (located at ERE1 −4099/−4096 bp and ERE2 −4087/−4084 bp) of the *LIFR* promoter in the presence of E2 (Fig. 6e, f), suggesting that nPAK4 regulates ERα+ breast cancer cell metastasis to the bone in part by targeting the expression of LIFR. LIFR overexpression (LIFR-Lv) was found to attenuate PAK4-mediated cell invasion in the presence of E2 (Fig. 6g). LIFR CRISPR/Cas9 KO increased the inhibited invasion of MCF-7 cells seen with knockdown PAK4 (Fig. 6h). These data support a necessary role of nPAK4/ER-mediated repression of LIFR. So, upregulated PAK4 and downregulated LIFR is a key step for ERα+ MCF-7 cells invasion with E2 treatment.

## Discussion

For the first time, we identified a role function of the nuclear PAK4 in promoting the breast-to-bone metastasis of ERα+ breast cancer via PAK4–ERα-LIFR axis. An analysis of human breast cancer samples with or without aggressive colonization of the bone revealed that nPAK4 expression was significantly associated with ERα+ as well as LIFR and breast cancer with bone metastasis that often leads to a poorer prognosis. In this regard, high nPAK4 expression may contribute to the aggressive phenotype in ERα+ breast cancer patients. In general, the PAK4 localization is mainly in the cytoplasm and perinucleus [25], and PAK4 plays crucial roles in a wide range of cellular processes [26], including promoting cancer cell invasion and migration [27–29]. By confocal imaging, we found that PAK4 and ERα entered the nucleus in breast cancer cell lines upon E2 stimulation. We demonstrated that PAK4 directly bind to the specific DNA-binding domain of ERα, which binds to cognate palindromic DNA sequences known as EREs. We showed that PAK4 decreased ER-induced transactivation in both *Drosophila* and mammalian cells, suggested an evolutionary conversation of this axis. Recently, experiments have indicated that PAK4 phosphorylated ERα-Ser305, and PAK4 may be a regulator of tamoxifen resistance by perturbing ERα signaling in breast cancer patients [30]. We suspect that the PAK4 phosphorylation of ERα may take place in the cytoplasm, and this phosphorylation is weaker than the PAK1-mediated ERα-Ser305 phosphorylation in breast cancer [31, 32]. Based on these findings, we hypothesize that: nPAK4 inhibits ERα-DNA-binding and reduces ERα-mediated transcriptional responses in an E2-dependent manner in ERα+ breast cancer patients.

The EMT is implicated in the progression of breast cancer. The loss of functional E-cadherin is a hallmark of EMT. Transcriptional repressors, including Snail, Slug, ZEB, Twist, and E47 inhibit E-cadherin expression, resulting in EMT and metastasis. In this study, we found that nPAK4, as a novel E-cadherin repressor, promoted EMT through the upregulation of Slug in breast cancer cells treated with E2. PAK4 is associated with a poor prognosis in various cancers and promotes migration and invasion [27, 33–35]. We also found in a nude mouse xenograft model that non-aggressive MCF-7 breast cancer cells with high bone metastatic potential expressed higher levels of PAK4 with E2 stimulation, which may enable these cells to alter the expression of downstream ERα target genes. More importantly, our data also point out that nPAK4 may be a key mediator of LIFR, an estrogen-regulated gene [36] important for maintaining cancer cells in a dormant state [18]. The loss of LIFR enhances invasion and downregulates dormancy, quiescence, and cancer stem cell-associated genes. The levels of LIFR are lower with bone metastases and are significantly and inversely correlated with patient outcomes in breast cancer patients [18]. We confirmed that LIFR is a downstream target gene of ERα and found that PAK4 and ERα bind to the LIFR promoter at a region containing two ERE half sites upstream of the translation start site. Together, these findings established that nPAK4 is a potent endogenous repressor of the ERα transactivation function and that nPAK4 may repress the expression of the ERα-induced gene LIFR in breast cancer cells, leading to bone metastasis. Importantly, PAK4 reversed nine of E2-mediated target genes expression by using RNA-seq analysis, including *CLSTN2*, *IL1R1*, *FOXM1*, *TNFRSF11B*, *CCNG2*, *TNS3*, *ABCG1*, *SMAD3*, and *SERPINA3*. Further studies will examine whether the target genes are involved in modulation of breast-bone metastasis in breast cancer by nPAK4 with E2 stimulation.

From the therapeutic standpoint, the notion that nPAK4 is relevant to the pathobiology of metastatic breast cancer is supported by the following line of evidence: First, altering the localization of PAK4 from the cytoplasm to the nucleus is the key step for ERα+ breast cancer metastasis in a hormone-dependent manner. Second, PAK4, as a negative regulator of ERα transactivation functions, might contribute to the aggressiveness of breast cancer cells by allowing the development of ERα-negative phenotypes. Furthermore, PAK4/ERα co-translocates to the nucleus and contributes to bone metastasis by targeting LIFR. Because PAK4 nuclear localization correlates with the development of ERα-negative phenotypes, leading to increased metastasis to the bone, searching for therapeutic strategies to block the entry of PAK4 into the nucleus would be useful for preventing breast cancer metastasis in ERα+ breast cancer. In conclusion, the findings presented here help to explain why the cancerous cells of a subset of patients with ERα+ breast cancer tend to spread to distant locations, and nPAK4 may be a novel biomarker molecule with potential value in clinical practice for predicting metastasis to the bone.

## Materials and methods

### Cell culture

MCF-7, ZR-75-1, and ZR-75-30 (Shanghai cell bank of CAS-Chinese Academy of Sciences) were cultivated in Minimum Essential Medium (MEM, Life, Shanghai, China) or RPMI-1640. Luciferase-expressing MCF-7-luc-F5 cells were originally purchased from Xenogen Corporation (Xenogen, Beijing, China) and were cultured in MEM (0.01 mg/ml insulin and 10% FBS). For estrogen treatment experiments, cells were maintained in phenol red-free MEM (5% dextran-charcoal-stripped fetal calf serum).

### Lentiviral production

PAK4-lentivirus (PAK4-Lv), PAK4 shRNA-lentivirus (shPAK4), and LIFR-lentivirus (LIFR-Lv) were from Shanghai GeneChem Company (GeneChem, Shanghai, China). The sequences of shPAK4 were described in our previously method [37].

### Plasmids

Flag-tagged ERα expression vectors were provided by Dr. Muyan M (Department of Biochemistry and Biophysics, University of Rochester Medical Center). GST-tagged ERα and deletions were sub-cloned in the pGEX-5x-2  (GE Healthcare, Waukesha, WI, USA) vectors. PAK4 full-length vectors were provided by Dr Audrey Minden (New Jersey, USA). GST-tagged PAK4 and delete mutations were inserted into pGEX-5x-1 backbone. si-PAK4 (sense 5′-CUUCAUCAAGAUUGGCGAGtt-3′) were designed and synthesized by Shanghai GeneChem Co., Ltd. Estrogen receptor β, ARE-luc, PRE-luc, ERE-luc, AR, and DHT was kindly provided by Dr Zhao Y. PRA and PRB expression vectors were kindly provided by Dr. P. Chambon. LIFR CRISPR/Cas9 KO Plasmid (h) was purchase from Santa Cruz Biotechnology (sc-400861).

### RNA isolation and real-time RT-PCR

The processes of RNA extraction and PCR are available under reference Li *et al*. [11]. The sequences of PCR primer are described in the Supplementary Table 5.

### Immunoblotting and immunoprecipitation

Immunoblotting and immunoprecipitation assays have been described previously [38]. Primary antibodies: ERα (Chemicon; Cell signaling; Santa Cruz Biotechnology), PAK4 (Cell Signaling), E-cadherin (BD Biosciences; Cell signaling), LIFR, PARP and α-Tubulin (Santa Cruz Biotechnology), c-Myc-tag and Flag-tag M2 (Sigma-Aldrich), His-tag and GFP-tag (GenScript Corporation), GAPDH (KangChen Bio-tech), Mbt (Boaosen Biotechnology Company). Secondary antibodies: Alexa Fluor 488- and 546 (Invitrogen, Carlsbad, CA, USA).

### Luciferase assay

PAK4/E2-mediated luciferase activities were detected in cells by using the Berthold Luminometer LB9570.

### GST pull-down assay

The equal amount of GST, GST-fusion proteins were combined with glutathione-conjugated Sepharose beads (Amersham Biosciences, Piscataway, NJ, USA)

### Immunofluorescence analysis

Immunofluorescence assay has been described previously in detail [39]. MCF-7 cells (treated with or without E2) or ERα positive breast cancer tissues were stained with PAK4 and ERα.

### *Drosophila* stocks and immunostaining of eye disc histology analysis

To establish overexpression of ERα in *Drosophila* eyes, ERα *Drosophila* line was crossed with *GMR-GAL4 Drosophila* line. *Mbt* and *Mbt*^*P1*^ mutant lines were the generous gifts of Raabe T. *Drosophila* eye disc analysis assay was mention in the previously described [40–42] and the *Drosophila* are fed with or without E2.

### ChIP and ChIP Re-IP

The experiments were performed essentially as described in the references [41, 43]. *LIFR* chromatin region (−4129 to −4034) was amplified with the following sequence: 5′-AATGGGGGTTTGTCCACACT-3′ and 5′-CCATACACAAGGGCTCAGGA-3′. *pS2* chromatin region (−3181 to −3062) was analyzed with the following sequence: 5′-GGGGTACTAAGGGACGACTGC-3′ and 5′-GCTGGAAACAGGGAAAGAAGG-3′. *GAPDH*: 5′-TACTAGCGGTTTTACGGGCG-3′ and 5′-TCGAACAGGAGCAGAGAGCGA-3′.

### RNA-seq analysis and DEGs

The total RNA extractions were using the TRIzol (Thermo Fisher Scientific, Shanghai, China). RNA-seq analysis was performed at Shanghai Kangcheng Bio-tech Inc. (Shanghai, China).

### Migration and invasion assays

The process of cancer cells migration and invasion has been previously described [44].

### Tail vein injections experimental metastasis model and bioluminescence

According to institutional and National Institutes of Health guidelines, the animal experiments were carried out. Female BALB/c Nude mice (weight < 20 g, *n* = 8/group, age 6–8 weeks) were randomly split into two groups. MCF-7-luc-F5 was infected with Con or PAK4-Lv and was inoculated into the tail vein of two group mice. BLI was measured in the IVIS™ 200 Imaging System. E2V (20 μg per dose, 0.1 μg/μl in H_2_O) were administered by oral gavage every day.

### Patient cohort and immunohistochemistry

All BMBC patients (*N* = 95) with no evidence of metastasis had confirmed primary breast cancer, the primary point was time to first recruited between 2003 and 2009. The secondary endpoints included overall survival and bone metastatic relapse. Several clinical examinations confirmed the occurrence of bone metastases, including MRI, CT, skeletal X-rays and isotopic bone scan. In addition, 187 NMBC samples were pathologically diagnosed as breast cancer by two experienced pathologists and none of the 187 patients had distant metastasis to the bone. All the enrolled patients with the diagnosis of breast cancer underwent breast resection or biopsy from the First Affiliated Hospital of China Medical University without having chemotherapy or radiation therapy. In total, 5 μm paraffin sections from formalin-fixed tissue were performed immunohistochemisty as previously described [11, 45]. The data and tissue collection were approved by the Human Research Ethics Committee of the First Affiliated Hospital of China Medical University.

### Statistical analysis

The SPSS software version 17.0 (Chicago, IL, USA) was used to analysis all statistical data. Kaplan-Meier overall survival curves demonstrated the survival differences between the positive and the negative nuclear expression of PAK4 patients. The univariate and multivariate Cox proportional models were used to calculate the hazards regression of patient overall survival. Two-sided Student’s *t* test was used to determinate the significant difference of luciferase data. *χ*^2^ test was applied to analyze the correlation between PAK4 and clinicopathological characteristics of immunohistochemistry.

## Electronic supplementary material

Supplementary figure S1

Supplementary figure S2

Supplementary figure S3

Supplementary figure S4

Supplementary figure legend

Supplementary table 1

Supplementary table 2

Supplementary table 3

Supplementary table 4

Supplementary table 5
