# Supplementary figures and images for: A mandatory role of nuclear PAK4-LIFR axis in breast-to-bone metastasis of ERα-positive breast cancer cells
Source: Oncogene. 2018 Sep 3;38(6):808–21. doi: 10.1038/s41388-018-0456-0 (PMC6367215; doi:10.1038/s41388-018-0456-0)

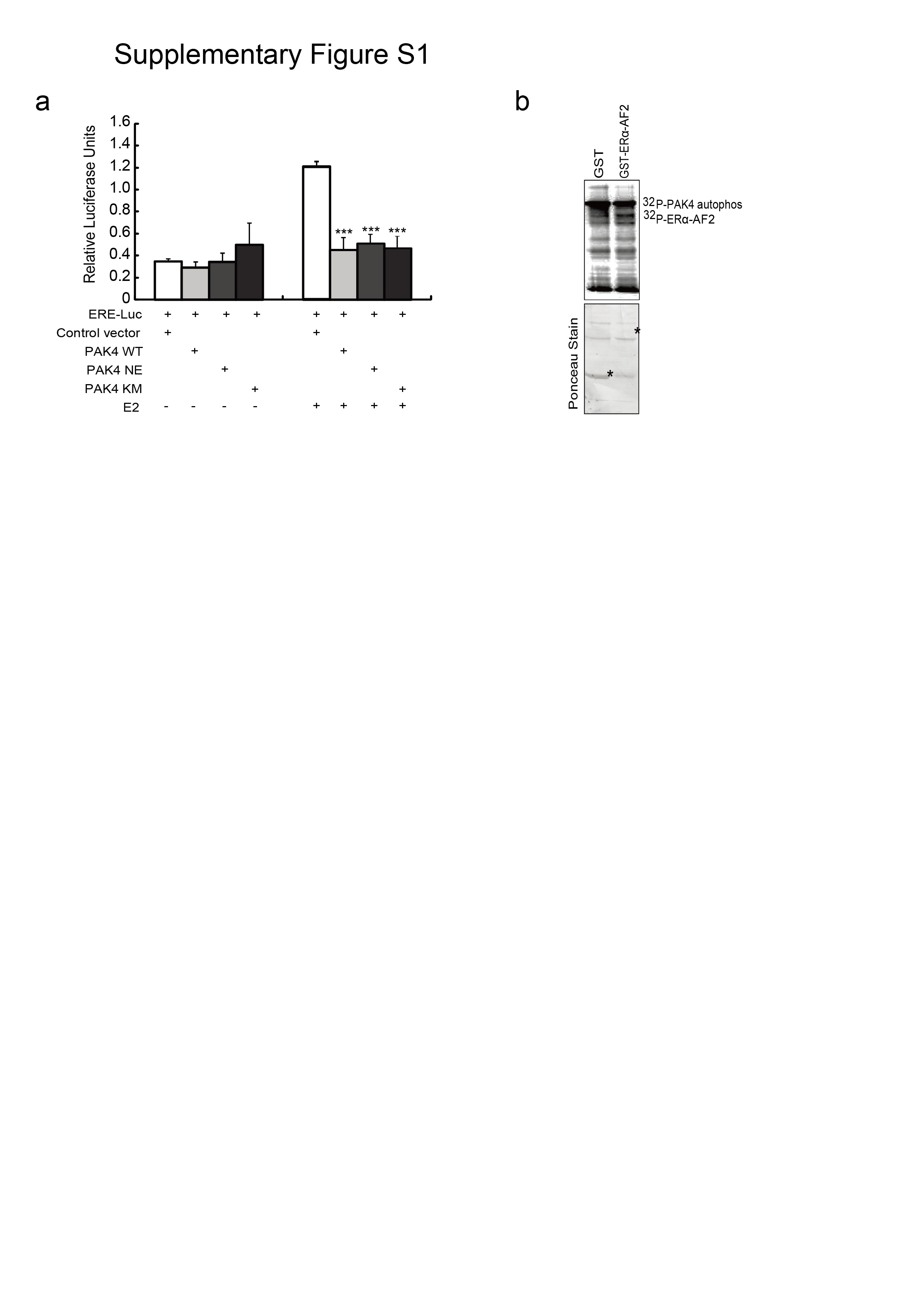

Supplement: Supplementary file 1 — Supplementary figure S1 [file 41388_2018_456_MOESM1_ESM.jpg]

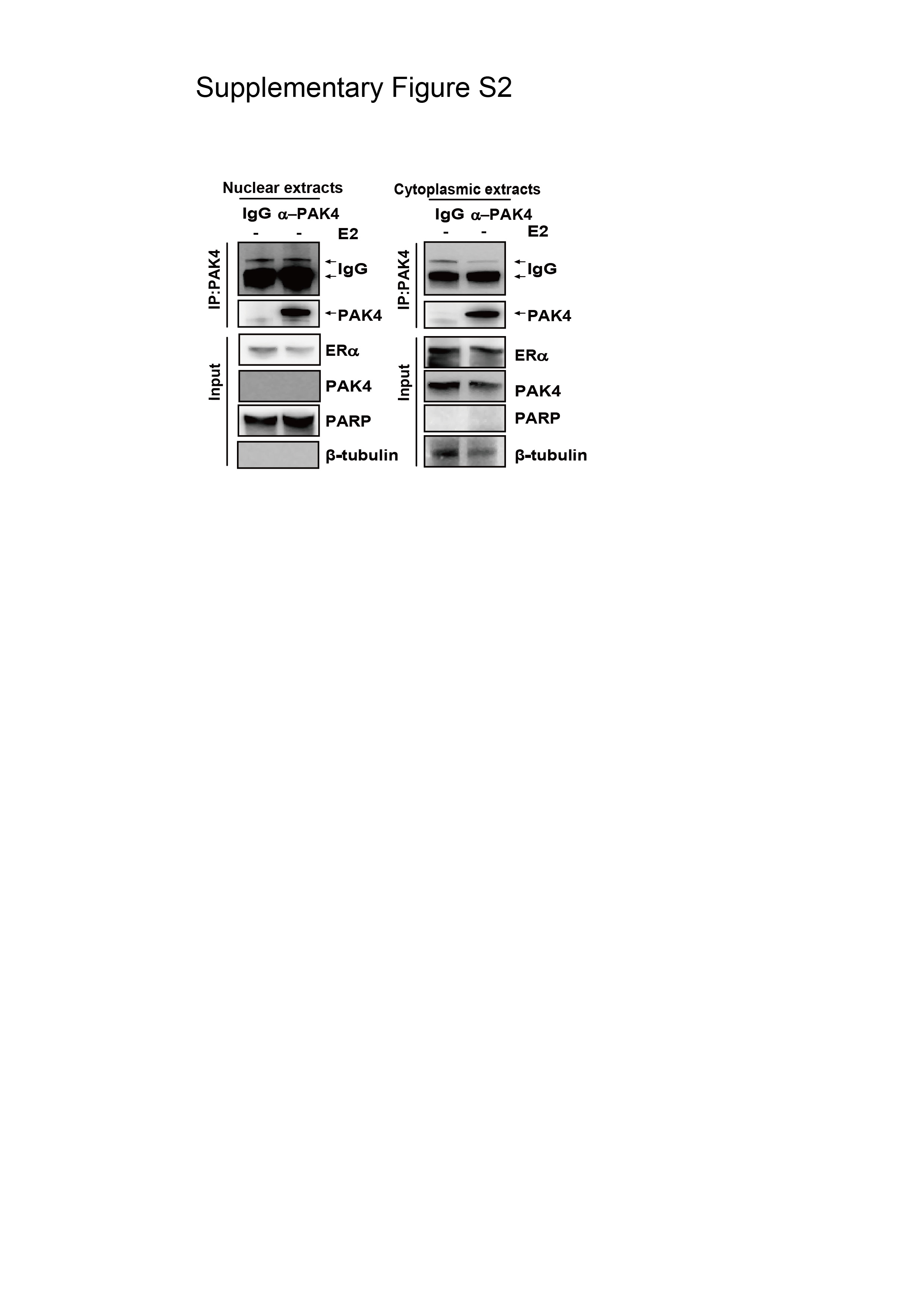

Supplement: Supplementary file 2 — Supplementary figure S2 [file 41388_2018_456_MOESM2_ESM.jpg]

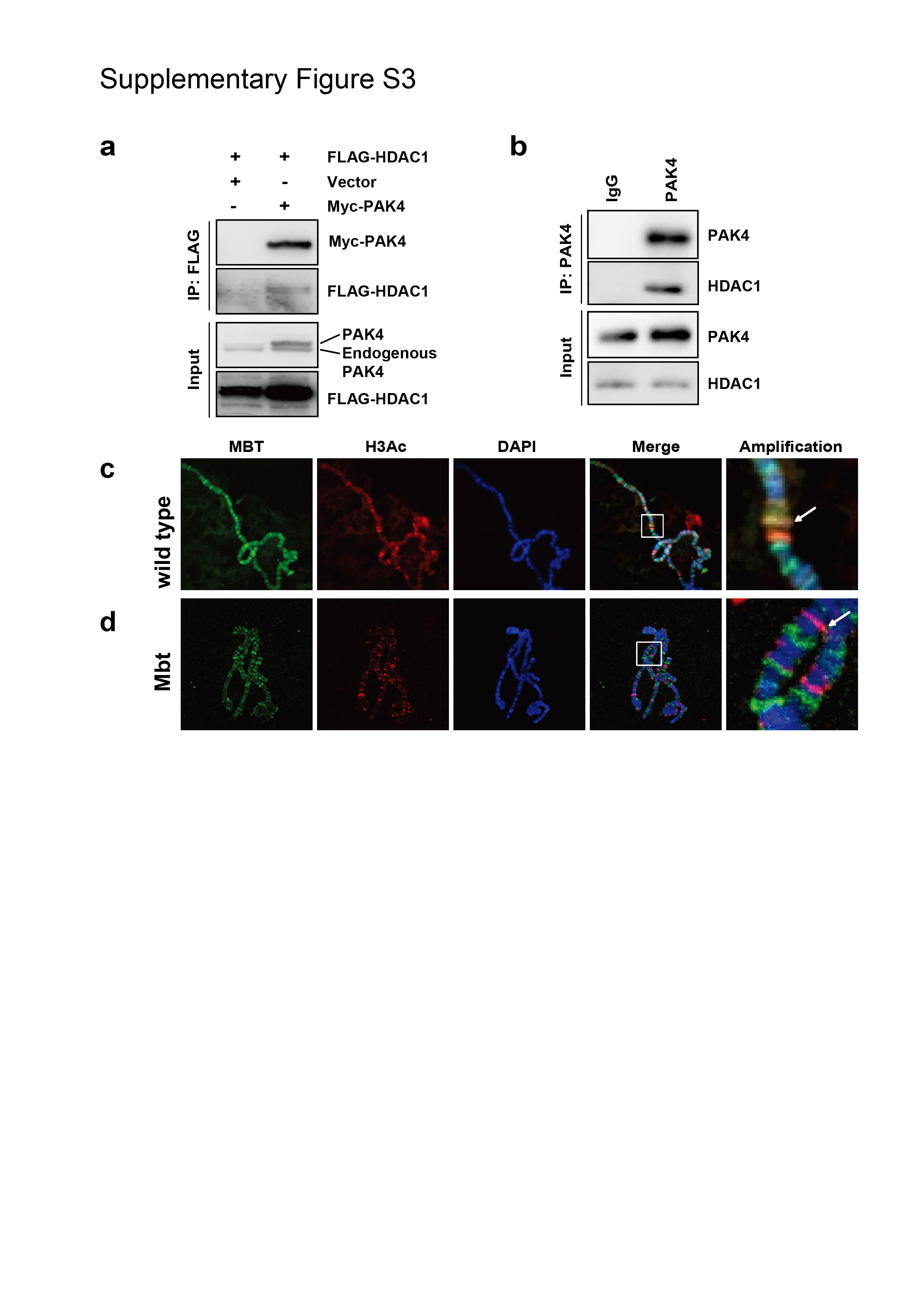

Supplement: Supplementary file 3 — Supplementary figure S3 [file 41388_2018_456_MOESM3_ESM.jpg]

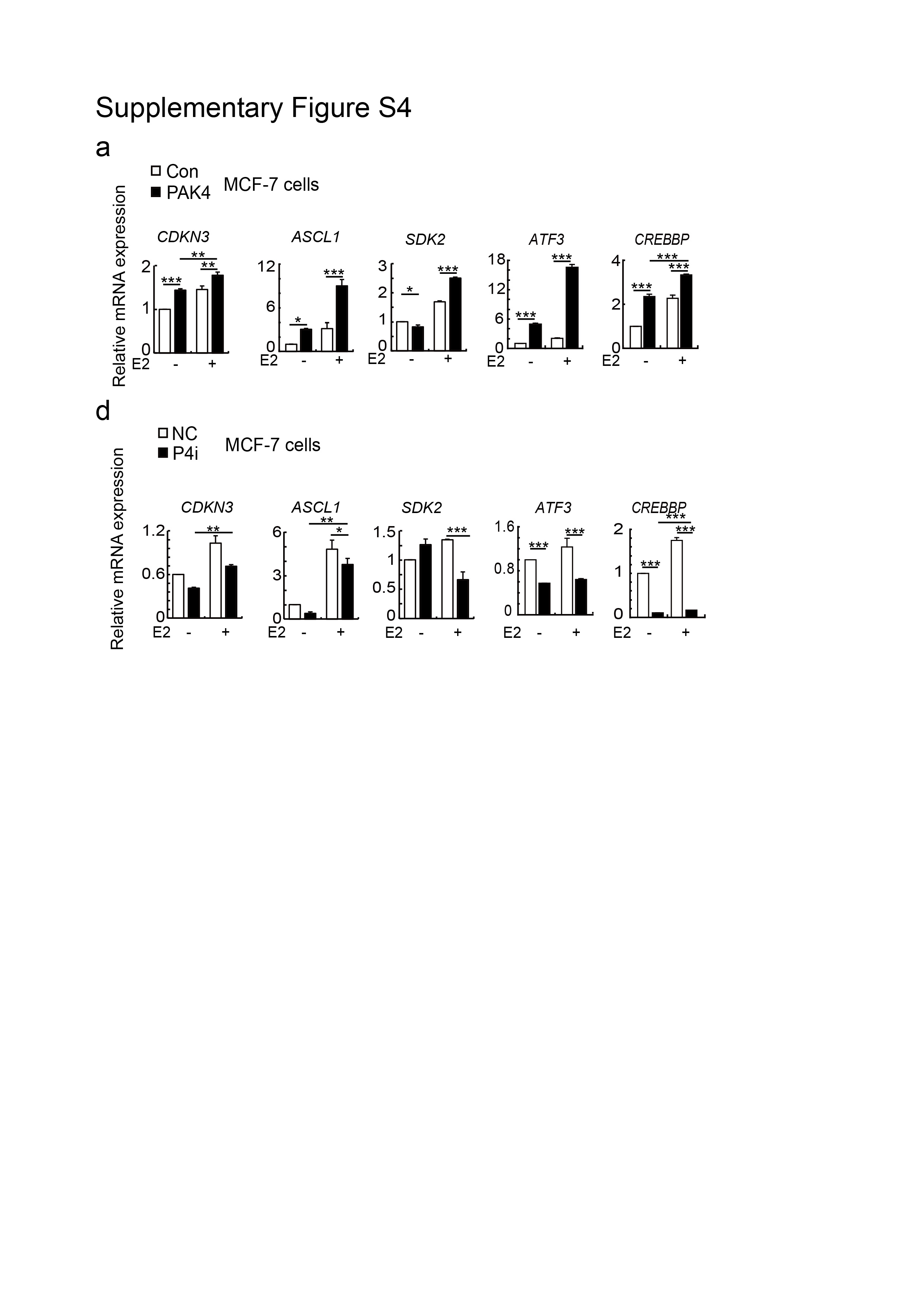

Supplement: Supplementary file 4 — Supplementary figure S4 [file 41388_2018_456_MOESM4_ESM.jpg]
