# Supplementary material for: A mandatory role of nuclear PAK4-LIFR axis in breast-to-bone metastasis of ERα-positive breast cancer cells
Source: Oncogene. 2018 Sep 3;38(6):808–21. doi: 10.1038/s41388-018-0456-0 (PMC6367215; doi:10.1038/s41388-018-0456-0)
Supplement: Supplementary file 5 — Supplementary figure legend [file 41388_2018_456_MOESM5_ESM.docx]

**Supplementary Figure S1**. PAK4-mediated ERE transcriptional activation did not require kinase activity. A. MCF-7 cells were co-transfected with the ERE-Luc together with PAK4 WT or PAK4 NE or PAK4 KM expression plasmid as indicated. Cells were treated with or without 10^-9^ M E2. The total amount of the transfected DNA was kept constant with the control vector. Error bars represent mean ± SD. * *P* < 0.05； ** *P* < 0.01; *** *P* < 0.001. b. PAK4 phosphorylated ERα AF2 domain. An in vitro kinase assay using purified GST, GST–ERα AF2 fusion proteins as substrates for PAK4 was performed. GST and GST–ERα AF2 were expressed and purified from Escherichia coli cells. Phosphorylations were detected with autoradiography. Black stars indicate the substrates.

**Supplementary Figure S2.** PAK4 and ERα were no physical interactions in the cytoplasmic and nuclear extracts from MCF-7 cells. MCF-7 cells were grown in phenol red-free MEM containing with 5% dextran-charcoal-stripped fetal calf serum for 72 h, Co-IP was performed from the nuclear and cytoplasmic fractions. β-tubulin and PARP were used as controls for the cytoplasmic and nuclear compartments, respectively.

**Supplementary Figure S3**. HDAC1 was an integral part of PAK4. a. HEK293 cells were transiently co-transfected with Myc-PAK4 and Flag-HDAC1. HEK293 cell extracts were Co-IP with α-Myc antibody or control IgG, and analysis by immunoblotting with anti-Myc and anti-Flag revealed the presence of PAK4 and HDAC1. b. The Co-IP detection of the interaction between PAK4 and HDAC1 in endogenous conditions. MCF-7 cell lysates were prepared and the PAK4 was immunoprecipitated with PAK4-specific antibody. Immunoblotting was done with anti-HDAC1 antibody (Proteintech, Wuhan, China) and anti-PAK4 antibody. c-d Polytene chromosomesfrom the third instarlarvae of fliescarrying wild type or UAS-Mbt and GMR-GAL4 expression plasmids were dissected. The location of UAS-Mbt on chromosomes was examined with anti-Mbt antibody (green). Polytene were stained with anti-H2Ac (red) and with DAPI to visualize DNA (blue).

**Supplementary Figure S4.** PAK4 up-regulated E2-mediated gene expression. a. MCF-7 cells with PAK4 stable over-expression were harvested after treatment with or without E2 (10^-9^ M) for 24 h. Total RNA was analyzed by Real-time quantitative PCR (RT-qPCR). b. MCF-7 cells with stable silencing PAK4 expression were harvested after treatment with or without E2 (10^-9^ M) for 24 h. Total RNA was analyzed by Real-time quantitative PCR (RT-qPCR). Error bars represent mean ± SD. * *P* < 0.05； ** *P* < 0.01; *** *P* < 0.001.
