# Supplementary material for: A mandatory role of nuclear PAK4-LIFR axis in breast-to-bone metastasis of ERα-positive breast cancer cells
Source: Oncogene. 2018 Sep 3;38(6):808–21. doi: 10.1038/s41388-018-0456-0 (PMC6367215; doi:10.1038/s41388-018-0456-0)
Supplement: Supplementary file 6 — Supplementary table 1 [file 41388_2018_456_MOESM6_ESM.doc]

**Supplementary Table 1**

**Correlation of nuclear PAK4 with clinicopathological variables in bone-metastasis breast cancer patients**

|  |  | **Nuclear PAK4** | |  |
| --- | --- | --- | --- | --- |
| **Clinicopathological characteristics** | **Cases(n=95)** | **Positive (n =54 )** | **Negative (n = 41)** | **p-Value** |
| **Age (year)** |  |  |  |  |
| **≤60**  **>60** | 75  20 | 40(42.1%)  14(14.7%) | 35(36.8%)  6(6.3%) | 0.181 |
| **ERα** |  |  |  |  |
| **positive**  **negative** | 57  38 | 38(40.0%)  16(16.8%) | 19(20.0%)  22(23.2%) | 0.018* |
| **PR** |  |  |  |  |
| **positive**  **negative** | 48  47 | 27(28.4%)  27(28.4%) | 21(22.1%)  20(21.1%) | 0.906 |
| **Her2** |  |  |  |  |
| **positive**  **negative** | 59  36 | 32(33.7%)  22(23.2%) | 27(28.4%)  14(14.7%) | 0.512 |
| **Ki-67** |  |  |  |  |
| **≤13%**  **>13%** | 39  56 | 24(25.2%)  30(31.6%) | 15(15.8%)  26(27.4%) | 0.441 |
