# Supplementary material for: A mandatory role of nuclear PAK4-LIFR axis in breast-to-bone metastasis of ERα-positive breast cancer cells
Source: Oncogene. 2018 Sep 3;38(6):808–21. doi: 10.1038/s41388-018-0456-0 (PMC6367215; doi:10.1038/s41388-018-0456-0)
Supplement: Supplementary file 7 — Supplementary table 2 [file 41388_2018_456_MOESM7_ESM.docx]

**Supplementary Table 2**

**Correlation of ERα and nuclear PAK4 expression in NMBC**

|  | **NMBC (187 cases)** | | | |
| --- | --- | --- | --- | --- |
| **Nuclear PAK4** | **Positive**  **67 (35.8%)** | **Negative**  **120 (64.2%)** | ***X*^2^** | ***P*** |
| **ERα** |  |  |  |  |
| **Positive** | **24 (12.8%)** | **70 (37.4%)** | **8.716** | **0.0032*** |
| **Negative** | **43 (23.0%)** | **50 (26.8%)** |  |  |
