# Supplementary material for: A mandatory role of nuclear PAK4-LIFR axis in breast-to-bone metastasis of ERα-positive breast cancer cells
Source: Oncogene. 2018 Sep 3;38(6):808–21. doi: 10.1038/s41388-018-0456-0 (PMC6367215; doi:10.1038/s41388-018-0456-0)
Supplement: Supplementary file 8 — Supplementary table 3 [file 41388_2018_456_MOESM8_ESM.doc]

**Supplementary Table 3**

**Univariate and multivariate analysis of nuclear PAK4 with regard to BMFS**

| **variable** | **HR** | **95% CI** | ***P* value** |
| --- | --- | --- | --- |
| **Univariate analysis** |  |  |  |
| Nuclear PAK4 |  |  |  |
| Negative | 1.00 |  |  |
| Positive | 2.223 | 1.336-3.698 | 0.002* |
| ERα |  |  |  |
| Negative | 1.00 |  |  |
| Positive | 0.958 | 0.738-1.244 | 0.748 |
| PR |  |  |  |
| Negative | 1.00 |  |  |
| Positive | 1.029 | 0.803-1.319 | 0.819 |
| Her2 |  |  |  |
| Negative | 1.00 |  |  |
| Positive | 1.063 | 0.886-1.276 | 0.510 |
| Ki-67 |  |  |  |
| ≤13% | 1.00 |  |  |
| >13% | 1.168 | 0.717-1.903 | 0.532 |
| **Multivariate analysis** |  |  |  |
| Nuclear PAK4 |  |  |  |
| Negative | 1.00 |  |  |
| Positive | 2.154 | 1.306-3.554 | 0.003* |

***BMFS*** bone metastasis-free survival, ***HR*** hazard ratio, ***CI*** confidence interval
