# Supplementary material for: A mandatory role of nuclear PAK4-LIFR axis in breast-to-bone metastasis of ERα-positive breast cancer cells
Source: Oncogene. 2018 Sep 3;38(6):808–21. doi: 10.1038/s41388-018-0456-0 (PMC6367215; doi:10.1038/s41388-018-0456-0)
Supplement: Supplementary file 9 — Supplementary table 4 [file 41388_2018_456_MOESM9_ESM.doc]

Supplementary Table 4. The overlapping nPAK4/ERα target genes.

| **Names** | **total** | **elements** |
| --- | --- | --- |
| GSE26834 GSE46924 P4i/NC | 1 | ABCG1 |
| GSE11352 GSE46924 P4i/NC | 1 | RET |
| GSE26834 GSE8597 P4i/NC | 1 | TNFRSF11B |
| GSE46924 P4i/NC | 2 | SYNPO2 SDK2 |
| GSE26834 P4i/NC | 2 | CLSTN2 SERPINA3 |
| GSE8597 P4i/NC | 1 | FOXM1 |
| GSE11352 P4i/NC | 4 | HSPB8 SLC7A5 SUSD3 ASCL1 |
