# Supplementary material for: A mandatory role of nuclear PAK4-LIFR axis in breast-to-bone metastasis of ERα-positive breast cancer cells
Source: Oncogene. 2018 Sep 3;38(6):808–21. doi: 10.1038/s41388-018-0456-0 (PMC6367215; doi:10.1038/s41388-018-0456-0)
Supplement: Supplementary file 10 — Supplementary table 5 [file 41388_2018_456_MOESM10_ESM.doc]

­­Supplementary Table 5. The specific PCR primers

| CCND1-F | CAGATCATCCGCAAACACGC | CCND1-R | AAGTTGTTGGGGCTCCTCAG |
| --- | --- | --- | --- |
| RARA-F | GTCAGGACAAGTCCTCAGGC | RARA-R | CCGGGTCACCTTGTTGATGA |
| TNFRSF11B-F | CCTCTGTGAAAACAGCGTGC | TNFRSF11B-R | AGGTGTCTTGGTCGCCATTT |
| CLSTN2-F | ACCGGGAGGCGAGAGC | CLSTN2-R | TCGATCCATGGCTTGTGCTT |
| SERPINA3-F | ATGGGAGATGCCCTTTGACC | SERPINA3-R | CATGGGCACCATTACCCACT |
| FOXM1-F | ATAGCAAGCGAGTCCGCATT | FOXM1-R | AGCAGCACTGATAAACAAAGAAAGA |
| ASCL1-F | CCAAGCAAGTCAAGCGACAG | ASCL1-R | TAGCCAAAGCCGCTGAAGTT |
| AP-2γ-F | GAAGAGGACTGCGAGGATCG | AP-2γ-R | GCTGATATTCGGCGACTCCA |
| SMAD3-F | AACGGGCAGGAGGAGAAATG | SMAD3-R | CTGGGGATGGTGATGCACTT |
| TNS3-F | AGTCAGCACAAAGGAGGACG | TNS3-R | CGATGGGGCTCTCTGACATC |
| CCNG2-F | GAAGGGGTCCAACTTCTCGG | CCNG2-R | ATCATTCTCCGGGGTAGCCT |
| IL1R1-F | AGGGATGACTACGTTGGGGA | IL1R1-R | CTCCAGCTCAAGCAGGACAA |
| CDKN3-F | GAGCAAGCCATAGACAGCCT | CDKN3-R | ATAATTGTATTGCTTGATGGTCTGT |
| SDK2-F | CTCAGGGAAGTGACAGCGAG | SDK2-R | CTGTTGGTGACGGTGGTGT |
| ABCG1-F | TGTCTGATGGCCGCTTTCTC | ABCG1-R | CTGGACACCACCTCATCCAC |
| ATF3-F | TTGCTAACCTGACGCCCTTT | ATF-R | GCTACCTCGGCTTTTGTGATG |
| CREBBP-F | CCCACAGACTTTGTGCTGCTATG | CREBBP-R | CCCACAGACTTTGTGCTGCTATG |
| PAK4-F | CCAGGATGAACGAGGAGCAG | PAK4-R | TAGGGAAGGCGGGAGATGAG |
| LIFR-F | GGTCAAATGCTTGTAAATGTAGCT G | LIFR-R | GCACATCACAACTGTTTATTCACC |
| SLUG-F | CAGCTCAGGAGCATACAG | SLUG-R | GAGGAGGTGTCAGATGGA |
| CDH1-F | CTGAGAACGAGGCTAACG | CDH1-R | GTCCACCATCATCATTCAATAT |
| β-actin-F | GGAAATCGTGCGTGACAT T | β-actin-R | CAGGCAGCTCGTAGCTCTT |
